# Supplementary material for: A Conway–Maxwell–Poisson-Binomial AR(1) Model for Bounded Time Series Data
Source: Entropy (Basel). 2023 Jan 7;25(1):126. doi: 10.3390/e25010126 (PMC9857646; doi:10.3390/e25010126)
Supplement: Supplementary file 1 [file entropy-25-00126-s001.zip › Metadaten_Geraete_Niederschlagshoehe_01981.html]

Stationsgeschichte der Messgeräte für Niederschlagshoehe


| Stationsgeschichte der Messgeräte für Niederschlagshoehe | | | | | | | | | |
| --- | --- | --- | --- | --- | --- | --- | --- | --- | --- |
| Stations\_ID | Stationsname | Geo. Laenge [Grad] | Geo. Breite [Grad] | Stations- hoehe [m] | Geberhoehe ueber Grund [m] | Von\_Datum | Bis\_Datum | Geraetetyp Name | Messverfahren |
| 1981 | Hamburg-Neuwiedenthal |  |  |  |  |  | 19620430 | Gerätetyp unbekannt | Niederschlagsmenge, konv. |
| 1981 | Hamburg-Neuwiedenthal | 9.9 | 53.48 | 2.5 | 1 | 19620501 | 19800626 | Hellmann | Niederschlagsmenge, konv. |
| 1981 | Hamburg-Neuwiedenthal | 9.9 | 53.48 | 2.5 | 1 | 19800627 | 20021023 | Hellmann | Niederschlagsmenge, konv. |
| 1981 | Hamburg-Neuwiedenthal | 9.9 | 53.48 | 2.5 | 1 | 20050215 | 20050228 | PLUVIO | Niederschlagsmenge, elektr. |
| 1981 | Hamburg-Neuwiedenthal | 9.9 | 53.48 | 3 | 1 | 20050301 |  | PLUVIO | Niederschlagsmenge, elektr. |

###### generiert: 06.03.2018 -- Deutscher Wetterdienst --
