# Supplementary material for: A Conway–Maxwell–Poisson-Binomial AR(1) Model for Bounded Time Series Data
Source: Entropy (Basel). 2023 Jan 7;25(1):126. doi: 10.3390/e25010126 (PMC9857646; doi:10.3390/e25010126)
Supplement: Supplementary file 1 [file entropy-25-00126-s001.zip › Metadaten_Parameter_nieder_tag_01981.html]

Datengewinnung --


| Datengewinnung | | | | | | | | | | | |
| --- | --- | --- | --- | --- | --- | --- | --- | --- | --- | --- | --- |
| Stations\_ID | Von\_Datum | Bis\_Datum | Stationsname | Parameter | Parameter- beschreibung | Einheit | Datenquelle (Strukturversion=SV) | Zusatz-Info | Besonderheiten | Literaturhinweis |
| 1981 | 19620501 | 20050228 | Hamburg-Neuwiedenthal | RS | tgl. Niederschlagshoehe Messnetz 6 | mm | Niederschlagsdaten aus Niederschlagsroutine des DWD oder Vorgängerdienste bis Einrichtung Nst(A) oder Nst(k) (1 Termin ca 7 MOZ und Tagessummen) | NBL:07:00-07:00 FT. GZ (bis 1990) sonst 07:30 - 07:30 FT. GZ |  |  |
| 1981 | 20050301 | 20180305 | Hamburg-Neuwiedenthal | RS | tgl. Niederschlagshoehe Messnetz 6 | mm | Niederschlagsdaten aus Niederschlagsroutine nach 01.04.2001 Terminwert und Tagessummen generiert aus SYNOP-Meldung des 06 UTC Termins | 05:51 - 05:50 FT. UTC |  |  |
| 1981 | 19620501 | 20050228 | Hamburg-Neuwiedenthal | RSF | Kennung fuer die Art RS (24-stdg.) Messnetz 6 | nummerischer Code | Niederschlagsdaten aus Niederschlagsroutine des DWD oder Vorgängerdienste bis Einrichtung Nst(A) oder Nst(k) (1 Termin ca 7 MOZ und Tagessummen) | NBL:07:00 GZ (bis 1990) sonst 07:30 GZ |  |  |
| 1981 | 20050301 | 20180305 | Hamburg-Neuwiedenthal | RSF | Kennung fuer die Art RS (24-stdg.) Messnetz 6 | nummerischer Code | Niederschlagsdaten aus Niederschlagsroutine nach 01.04.2001 Terminwert und Tagessummen generiert aus SYNOP-Meldung des 06 UTC Termins | 05:50 UTC |  |  |
| 1981 | 19620501 | 20050228 | Hamburg-Neuwiedenthal | SH\_TAG | Schneehoehe Tageswert | cm | Niederschlagsdaten aus Niederschlagsroutine des DWD oder Vorgängerdienste bis Einrichtung Nst(A) oder Nst(k) (1 Termin ca 7 MOZ und Tagessummen) | NBL:07:00 GZ (bis 1990) sonst 07:30 GZ |  |  |
| 1981 | 20050301 | 20180305 | Hamburg-Neuwiedenthal | SH\_TAG | Schneehoehe Tageswert | cm | Niederschlagsdaten aus Niederschlagsroutine nach 01.04.2001 Terminwert und Tagessummen generiert aus SYNOP-Meldung des 06 UTC Termins | 05:50 UTC |  |  |

###### Legende: FT = Folgetag; GZ = Gesetzliche Zeit

###### generiert: 06.03.2018 -- Deutscher Wetterdienst --
