# Supplementary material for: A Conway–Maxwell–Poisson-Binomial AR(1) Model for Bounded Time Series Data
Source: Entropy (Basel). 2023 Jan 7;25(1):126. doi: 10.3390/e25010126 (PMC9857646; doi:10.3390/e25010126)
Supplement: Supplementary file 1 [file entropy-25-00126-s001.zip › Metadaten_Stationsname_01981.html]

Geschichte der Stationsnamen --


| Geschichte der Stationsnamen | | | | | | | | | | | |
| --- | --- | --- | --- | --- | --- | --- | --- | --- | --- | --- | --- |
| Stations\_ID | Stationsname | Von\_Datum | Bis\_Datum |
| 1981 | Hamburg-Neuwiedenthal | 19430301 |  |
